# Cyclin E2 is the predominant E-cyclin associated with NPAT in breast cancer cells

**DOI:** 10.1186/s13008-015-0007-9

**Published:** 2015-02-19

**Authors:** Samuel Rogers, Brian S Gloss, Christine S Lee, Claudio Marcelo Sergio, Marcel E Dinger, Elizabeth A Musgrove, Andrew Burgess, Catherine Elizabeth Caldon

**Affiliations:** The Kinghorn Cancer Centre and Cancer Research Program, Garvan Institute of Medical Research, Sydney, NSW Australia; St Vincent’s Clinical School, Faculty of Medicine UNSW, Sydney, Australia; Wolfson Wohl Cancer Research Centre, University of Glasgow, Garscube Estate, Glasgow, G61 1QH UK

**Keywords:** Cyclin E1, Cyclin E2, CDK2, Centrosome, Cajal bodies, Histone Locus bodies (HLB), Spliceosomes, NPAT, Histones, Breast cancer

## Abstract

**Background:**

The cyclin E oncogene activates CDK2 to drive cells from G_1_ to S phase of the cell cycle to commence DNA replication. It coordinates essential cellular functions with the cell cycle including histone biogenesis, splicing, centrosome duplication and origin firing for DNA replication. The two E-cyclins, E1 and E2, are assumed to act interchangeably in these functions. However recent reports have identified unique functions for cyclins E1 and E2 in different tissues, and particularly in breast cancer.

**Findings:**

Cyclins E1 and E2 localise to distinct foci in breast cancer cells as well as co-localising within the cell. Both E-cyclins are found in complex with CDK2, at centrosomes and with the splicing machinery in nuclear speckles. However cyclin E2 uniquely co-localises with NPAT, the main activator of cell-cycle regulated histone transcription. Increased cyclin E2, but not cyclin E1, expression is associated with high expression of replication-dependent histones in breast cancers.

**Conclusions:**

The preferential localisation of cyclin E1 or cyclin E2 to distinct foci indicates that each E-cyclin has unique roles. Cyclin E2 uniquely interacts with NPAT in breast cancer cells, and is associated with higher levels of histones in breast cancer. This could explain the unique correlations of high cyclin E2 expression with poor outcome and genomic instability in breast cancer.

**Electronic supplementary material:**

The online version of this article (doi:10.1186/s13008-015-0007-9) contains supplementary material, which is available to authorized users.

## Findings

The canonical function of cyclin E is the activation of CDK2 (cyclin dependent kinase 2) to phosphorylate Rb, hence promoting the release of E2F transcription factors and progression of the cell cycle from G_1_ to S phase [1]. However there are other functions for cyclin E that may be CDK2 dependent or independent, including transcriptional processing, origin firing, and centrosome duplication [2]. The wide range of cyclin E functions may explain the necessity for two cyclin E proteins: E1 and E2. Both these proteins activate CDK2, but are encoded by genes on different chromosomes (cyclin E1: CCNE1 at 19q12; cyclin E2: CCNE2 at 8q22.1). Cyclin E1 and E2 have differences in tissue expression, transcription and post-transcriptional regulation, and have distinct affinities for other proteins, e.g. p107 [1,3]. In this study we examined the localisation of cyclin E1 and E2 and report unique sites of localisation in breast cancer cells.

**Figure 1:**
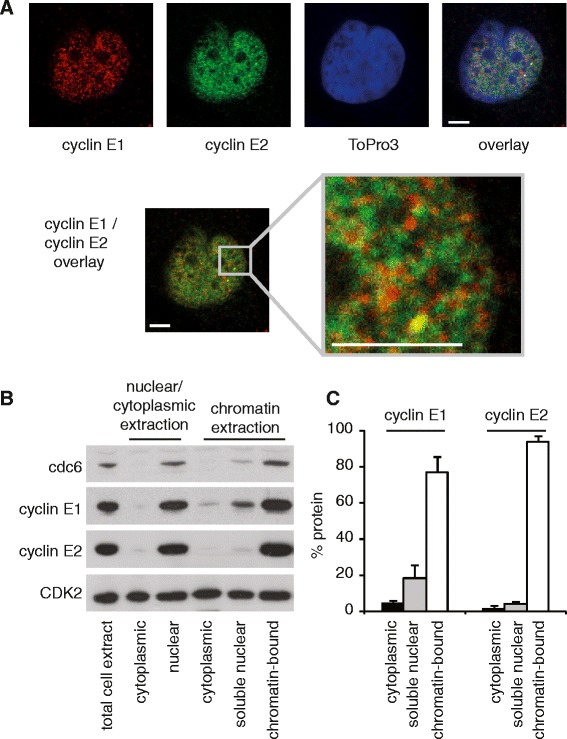
**Cyclins E1 and E2 localise to unique foci, and have distinct subcellular distribution. A**. Confocal images of T-47D breast cancer cells immunoprobed with cyclin E1 (red) or cyclin E2 (green), and counterstained with ToPro3 (blue, nuclei). Inset at higher magnification. Scale bars = 5 μm. Experiments are performed in triplicate. Similar data obtained in MCF-7 cells are shown in Additional file 1. **B**. T-47D cells were lysed to extract total cell proteins (lane 1), total nuclear (lane 2) and total cytoplasmic (lane 3) lysates. In parallel, cell lysates were purified to extract soluble cytoplasmic proteins, soluble nuclear proteins, and chromatin bound proteins. PAGE separated proteins were western blotted for Cdc6 (predominantly chromatin bound), CDK2 (cytoplasmic, nuclear and chromatin bound), cyclin E1 and cyclin E2. **C**. Cyclins E1 and E2 were quantitated from duplicate experiments using densitometry (ImageJ), and soluble cytoplasmic, soluble nuclear, and chromatin-bound fractions graphed as a percentage of total extracted protein. Error bars show range.

We previously identified that cyclin E1 and E2 are expressed in different cell line subpopulations due to distinct cell cycle regulation [4]. Close examination revealed that cyclin E1 and E2 localise to unique foci within the nucleus of T-47D and MCF-7 breast cancer cells (Figure 1A and Additional file 1). Several large bright foci exclusively localised with either cyclin E1 or E2, while some foci showed co-localisation (Figure 1A, inset, and Additional file 1, inset).

Cyclins E1 and E2 have cytoplasmic, nuclear and chromatin associated functions [1,2]. Cell fractionation showed that both cyclin E1 and E2 were predominantly nuclear and a large proportion was extracted with chromatin (Figure 1B). However a significant proportion of cyclin E1 was nucleolar and not chromatin associated (18.5%) compared to a smaller proportion of cyclin E2 (4.2%), and both proteins occurred at only very low levels in the cytoplasm (Figure 1B). Thus the majority of cyclin E1 and E2 is located on chromatin, but there is a small but significant proportion of cyclin E1 that is localized to non-chromatin foci.

**Figure 2:**
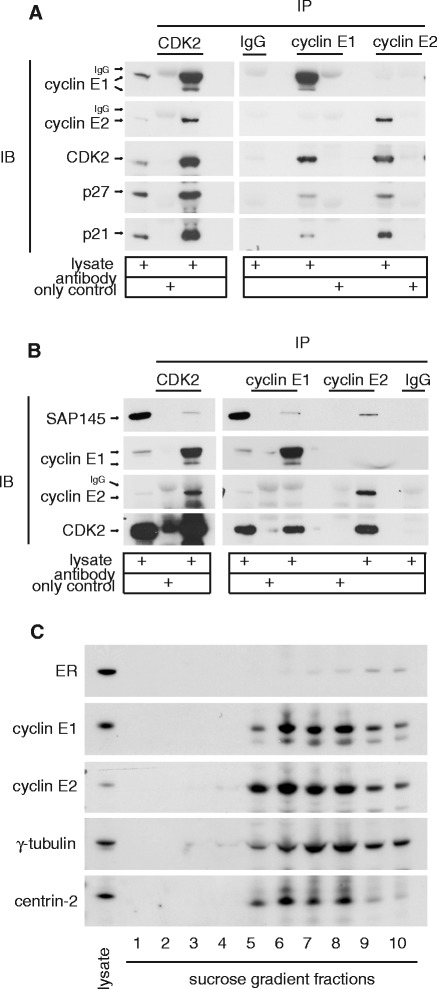
**Common functional complexes of cyclin E1 and E2. A**. Cyclin E1 and E2 both co-immunoprecipitate CDK2/CDK2 inhibitor complexes. Lysates of T-47D cells were immunoprecipitated and then western blotted using the indicated antibodies. Data are representative of duplicate experiments. Similar data from MCF7 cells are shown in [19]. IB: immunoblot; IP: immunoprecipitation **B**. Cyclin E1 and E2 both co-immunoprecipitate SAP145. Lysates of T-47D cells were immunoprecipitated and then western blotted using the indicated antibodies. Data are representative of triplicate experiments. In A. and B. arrows indicate protein of interest; IgG is non-specific immunoglobulin G staining. **C**. Cyclins E1 and E2 both co-purify with centrosomes. T-47D cells were arrested and synchronised at G_0_ with anti-estrogen ICI 182780 followed by estrogen stimulation for 16h. Lysates were separated by ultracentrifugation on sucrose gradients, fractionated, then pelleted and resuspended in sample buffer for western blotting with the indicated antibodies. γ-tubulin and centrin-2 are centrosome components, and estrogen receptor α (ER) is a non-centrosomal negative control. Data are representative of duplicate experiments. Similar data obtained in MCF-7 cells are shown in Additional file 2.

We next examined a range of cyclin E functions to determine if unique localisation of cyclin E1 or E2 was associated with a unique function. Cyclin E binds and activates CDK2, and this activity is inhibited by CDK inhibitors p21^Waf1/Cip1^ and p27^Kip1^. Both cyclin E1 and E2 form cyclin/CDK2/CDK inhibitor complexes, although these complexes are mutually exclusive (Figure 2A). Cyclin E/CDK2 phosphorylates splicing complexes which may coordinate pre-mRNA splicing with the G_1_/S transition [5]. These functional complexes appear common to cyclin E1 and cyclin E2, as in T-47D cells both proteins co-immunoprecipitate a major component of the spliceosome, SAP 145 (Figure 2B). Centrosomes are major cytoplasmic bodies located at the nuclear periphery. We identified that both cyclins E1 and E2 were localised to the centrosome complexes of T-47D and MCF-7 breast cancer cells using sucrose gradient fractionation of centrosomes and western blotting (Figure 2C, and Additional file 2), consistent with previous data showing specific localisation of cyclin E1 to centrosomes by immunofluorescence [6].

**Figure 3:**
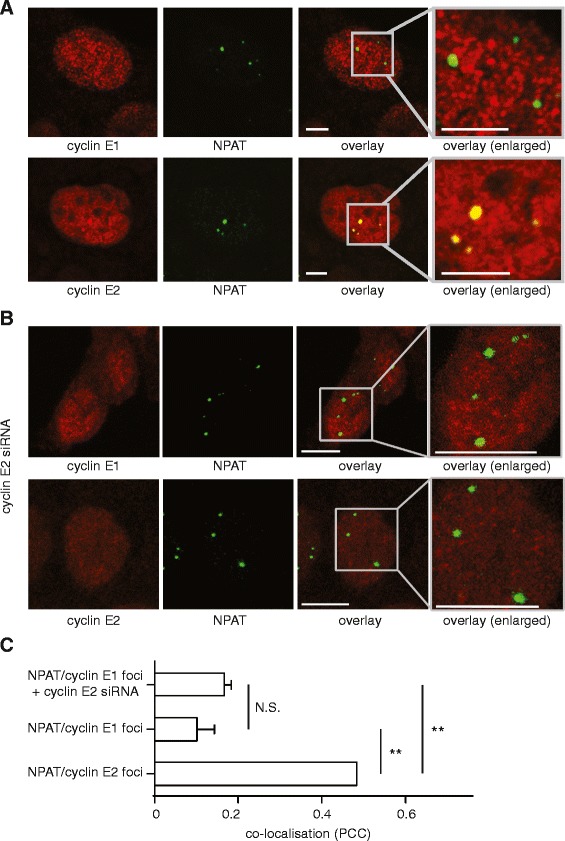
**Cyclin E2, but not cyclin E1, co-localises with NPAT by immunofluorescence in breast cancer cells. A**. Cyclin E2 localises to NPAT foci. Confocal images of T-47D cells immunoprobed with cyclin E1 or cyclin E2 (red) and NPAT (green). Experiments performed in triplicate. Example of lack of co-localisation of cyclin E1 (antibody: HE12) and NPAT (antibody: C-19) is shown, and is representative of similar data with cyclin E1 (antibody: Epitomics) and NPAT (antibody: 27) co-staining (not shown). Scale bars = 5μm. Similar data obtained in MCF-7 cells are shown in Additional file 3. **B**. Confocal images of T-47D cells treated with 20nM cyclin E2 siRNA for 48h, and then immunoprobed with cyclin E1 or cyclin E2 (red) and NPAT (green). Scale bars = 10μm. **C**. Quantitation of co-localisation using Pearson's correlation coefficient (PCC) which quantifies positional relationship from confocal images on a scale of -1 to +1. Statistical significance was calculated with one-way ANOVA and Tukey’s multiple comparisons, where N.S. indicates not significant and ** indicates P < 0.01. Data pooled from duplicate experiments. Similar data obtained in MCF-7 cells are shown in Additional file 3.

Cyclin E directly coordinates histone gene transcription with G_1_ to S phase transition via the phosphorylation of histone transcription factor NPAT in the Histone Locus Bodies (HLB) which localise to histone gene clusters on chromosomes 1 and 6 [7-9]. We found by immunofluorescence that cyclin E2 co-localised with the major HLB protein, NPAT, in T-47D (Figure 3A) and MCF-7 breast cancer cells (Additional file 3), but NPAT rarely co-localised with cyclin E1. The strong association between cyclin E2 and NPAT may be due to the relatively high levels of cyclin E2 observed in breast cancer cell lines [4]. However we observe that cyclin E1 does not relocalise to NPAT foci upon cyclin E2 siRNA treatment (Figure 3B and C). This suggests that the specific cyclin E2-NPAT interaction is due to intrinsic features of cyclin E2 rather than excess cyclin E2 preventing an interaction between cyclin E1 and NPAT.

**Figure 4:**
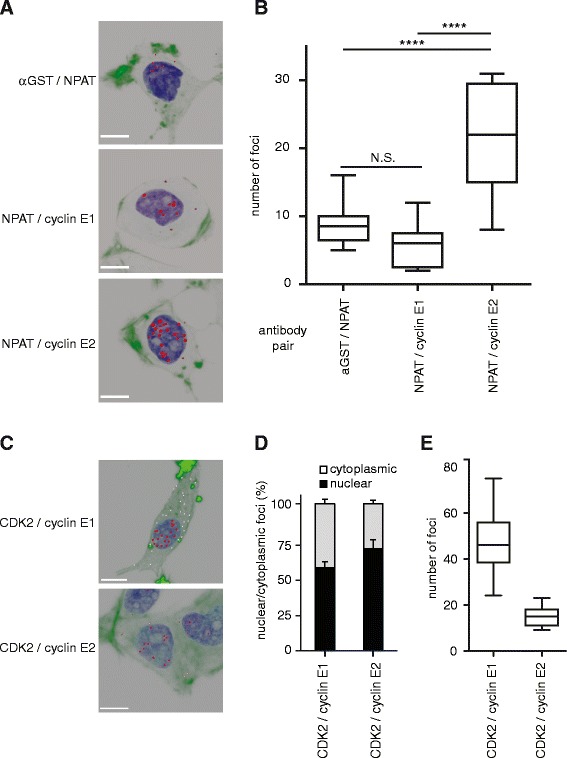
**Cyclin E2, but not cyclin E1, co-localises with NPAT in T-47D cells by PLA. A**. Proximity Ligation Assay (PLA) for cyclin E1/NPAT (antibodies: cyclin E1 – Epitomics; NPAT – 27) and cyclin E2/NPAT (antibodies: cyclin E2 – Epitomics; NPAT – 27). Images are 3-D rendered serially stacked confocal images assembled with Imaris software. NPAT/αGST staining was performed as a negative control (antibodies: NPAT – 27, αGST – [23]). Representative cells are shown, scale bars = 10μm. **B**. Quantitation of A. where number of foci were quantitated from 10-15 cells per antibody pair. Statistical significance was calculated with one-way ANOVA and Tukey’s multiple comparisons, where N.S. indicates not significant and **** indicates P < 0.0001. Data pooled from duplicate experiments. **C**. Cyclin E1/CDK2 (cyclin E1- HE12, CDK2 – M2) and cyclin E2/CDK2 (cyclin E2 – Epitomics, CDK2 – D12) PLA were performed as positive controls. Representative cells are shown with nuclear foci pseudocoloured in red, and cytoplasmic foci pseudocoloured in white. Scale bars = 10μm. **D**.**/E**. Quantitation of C. including relative nuclear/cytoplasmic foci **(D.)** and total foci **(E.)**. Data pooled from duplicate experiments.

We confirmed our findings using the *in situ* Proximity Ligation Assay (PLA), which detects the co-localisation of two antibodies within 40nm on fixed cells by PCR amplification of a linker probe. PLA analysis identified an average of 22 nuclear NPAT-E2 foci per cell, consistent with the multiple HLBs which are detected in aneuploid cancer cell lines [10] (Figure 4A). NPAT-cyclin E2 interactions were 4-fold higher than the number of cyclin E1-NPAT interactions (P < 0.0001; Figure 4B). Cyclin E1-NPAT interactions did not exceed background levels of the αGST/NPAT negative control, and hence are unlikely to represent true HLBs (Figure 4B). Together the immunofluorescence and PLA data indicate that cyclin E2 is the major E-cyclin within HLBs in breast cancer cells and is likely to have a particular role in coordinating the cell cycle with histone transcription.

As a positive control for PLA analysis we examined cyclin E1-CDK2 and cyclin E2-CDK2 interactions. We observed that both cyclin E1 and cyclin E2 had predominantly nuclear interactions with CDK2 (Figure 4C and D). A proportion of both cyclin E1-CDK2 and cyclin E2-CDK2 foci were cytoplasmic (Figure 4C and D) which is consistent with nuclear-cytoplasmic shuttling of these complexes [11]. Cyclin E1-CDK2 interactions were 2-fold more abundant than cyclin E2-CDK2 (Figure 4E), which again suggests that it is unlikely that excess cyclin E2 prevents cyclin E1 from interacting with other binding partners such as NPAT.

Previous publications describe binding of “cyclin E” to NPAT, whereas we here identify that cyclin E2 is the major E-cyclin within HLBs in breast cancer cells. The previous studies were performed prior to the development of specific cyclin E1 and E2 antibodies, and relied upon the cyclin E HE67 (cyclin E1 aa366-381) and HE11 (full-length protein) antibodies which are raised using epitopes that may not effectively discriminate cyclin E1 and cyclin E2 [8,9]. While cyclin E1 may not influence histone transcription in breast cells via NPAT it could influence it via other pathways. Cyclin E/CDK2 indirectly controls histone transcription via E2F-mediated transcription of NPAT [12], and by phosphorylation of the HIRA protein which is a repressor of histone transcription that operates outside S phase [13].

**Figure 5:**
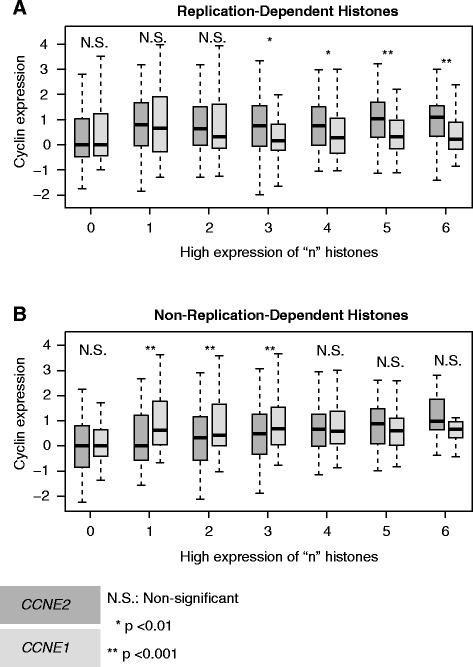
**Increased Cyclin E2 expression is associated with higher levels of replication-dependent histones in breast cancers.** Box plots illustrate the change in mRNA expression levels of *CCNE2* compared to *CCNE1* as replication-dependent **(A.)** and non-replication-dependent **(B.)** histone expression increases in 526 breast cancer samples. Breast cancer samples were grouped according to the number of replication dependent and independent histones displaying above median expression. Gene expression was normalized to the median expression of group 0 for each sample. p-values were calculated using a Mann-Whitney-U test. Boxes represent the normalized median expression and the 1^st^ and 3^rd^ quartiles and whiskers extend 1.5x the IQR from median.

Our observation of a specific NPAT-cyclin E2 interaction in breast cancer cell lines was supported by our findings of high expression of replication-dependent histones in breast cancers that have high expression of cyclin E2. We examined the transcript profiles of breast cancers from The Cancer Genome Atlas (TCGA) for cyclin E and histone expression. In 526 breast cancers, high CCNE2 expression is associated with high levels of replication-dependent histones that are under the control of NPAT (Figure 5A). However this pattern is not observed for CCNE1 (Figure 5A), nor with non-replication dependent histones (Figure 5B).

Cyclin E1 has been recognised as an important oncogene for 20 years [14]. The high degree of sequence homology between cyclin E1 and E2 suggests that many of their functions may be interchangeable, but recent publications in cancer and liver biology show that these proteins have unique regulation and function [15,16]. Our re-examination of cyclin E function has identified that cyclin E2 is likely to have particular role in histone regulation in breast cancer via its unique interaction with NPAT. Cyclin E2 has a strong prognostic role in breast cancer [15], and induces genomic instability that is associated with defects in chromosome condensation [3]. This could be in part due to excessive histone production, as disruption of histone equilibrium is a predicted cause of genomic instability [17].

Our identification of multiple foci that contained only cyclin E1 or E2 indicates that there are other unique interactions. This is not surprising given that the low molecular weight derivatives of cyclin E1 also has unique binding and function in cancer cells compared to the full length protein [18]. Future studies should carefully differentiate cyclin E1 and E2 and their isoforms, especially since each protein has unique expression patterns and their expression has distinct correlation with patient outcome in cancer [1].

## Methods

### Cell lines

Cell lines were authenticated by STR profiling (CellBank Australia, Westmead, NSW, Australia) and cultured for <6 months after authentication. Cyclin E1 and E2 siRNA treatment was performed and validated by western blotting as described in [19].

### Immunoblotting and immunoprecipitation

Collection of whole cell lysates [20], chromatin [21] and sucrose gradient fractions of centrosomes [22] were performed as described. Lysates were separated using NuPage polyacrylamide gels (Invitrogen) prior to transfer to PVDF membranes. Western blotting, immunofluorescence and PLA antibodies are: Cdc6 (180.2), CDK2 (M2, D12), centrin-2 (S-19), cyclin E1 (HE12), estrogen receptor α (HC20), NPAT (C-19, 27), SAP145 (A-20), γ-tubulin (C-11) (Santa Cruz Biotechnology); cyclin E2 (Epitomics); p21 (610234) and p27 (610242) (BD Biosciences); αGST [23]. Immunoprecipitation antibodies are: CDK2 (C-19), cyclin E1 (C-19), NPAT (C-19, 27), non-immune IgG (Santa Cruz Biotechnology), and cyclin E2 (Epitomics). Specificity of cyclin E1 and E2 antibodies was demonstrated in [15,19]. Additionally, we show specific loss of cyclin E1 and cyclin E2 immunofluorescence signal with siRNA treatment to cyclin E1 (Additional file 4) and cyclin E2 (Figure 3).

### Immunofluorescence and microscopy

Cells were fixed with 4% PFA/PBS for 20 min at room temperature, with or without methanol post-fixation ( -20°C for 20 min). Samples were blocked with 1% BSA/PBS, stained with the indicated antibodies and counterstained with ToPro3/DAPI (Jackson ImmunoResearch Laboratories). Co-localisation was quantitated by detecting overlapping pixels with Imaris v8.0 (Bitplane) and analysed with Pearson’s Correlation Coefficient [24]. For PLA, PFA fixed cells were subjected to the Duolink Proximity Ligation Assay (Sigma) as described by the manufacturer. Confocal microscopy was performed on Leica DMRBE/DMIRE2. Images were analysed with Imaris where individual spots were defined with a variable and initial size estimate of 0.5 μm. Images were processed with Adobe Photoshop, and adjusted for optimal brightness/contrast. Minimal gamma changes were made to enable visualisation of overlaid signals.

### Bioinformatics

Expression values in 526 breast cancer samples of *CCNE1*, *CCNE2* and representative replication-dependent and -independent histones (Additional file 5) were accessed from the cBioPortal [25] using the CGDSR package [26] in R [27]. For each sample the number of histones with high expression (> median across patients) was established for histone subsets. Samples were grouped according to the number of histones having above median expression. For each group, the expression level of CCNE1 and CCNE2 was normalised to 100% of the median expression in the patient group with zero highly expressed histones.

## Additional files

Additional file 1:
**Cyclins E1 and E2 localise to distinct nuclear foci in MCF-7 cells.** A. Confocal images of MCF-7 breast cancer cells immunoprobed with cyclin E1 (red) or cyclin E2 (green), and counterstained with ToPro3 (blue, nuclei). Inset at higher magnification. Scale bars = 5 μm. Experiments are performed in triplicate. Additional file 2:
**Cyclins E1 and E2 co-sediment with centrosome components in MCF-7 cells.** A. Cyclins E1 and E2 both co-purify with centrosomes. MCF-7 cells were arrested and synchronised at G_0_ with anti-estrogen ICI 182780 followed by estrogen stimulation for 16h. Lysates were separated by ultracentrifugation on sucrose gradients, fractionated, then pelleted and resuspended in sample buffer for western blotting with the indicated antibodies. γ-tubulin and centrin-2 are centrosome components, and Grb2 and estrogen receptor α (ER) are non-centrosomal negative controls. Data are representative of duplicate experiments. Additional file 3:
**Cyclin E2, but not cyclin E1, co-localises with NPAT by immunofluorescence in MCF-7 cells.** A. Confocal images of MCF-7 cells immunoprobed with cyclin E1 or cyclin E2 (red) and NPAT (green). Experiments performed in duplicate. Scale bars = 10μm. B. MCF-7 cells were treated with 20nM cyclin E2 siRNA for 48h as described in [19]. Co-localisation of cyclin E1 or cyclin E2 with NPAT using Pearson's correlation coefficient (PCC) which quantifies positional relationship from confocal images on a scale of -1 to +1. Statistical significance was calculated with one-way ANOVA and Tukey’s multiple comparisons, where N.S. indicates not significant and ** indicates P < 0.01. Data pooled from duplicate experiments. Additional file 4:
**Specific immunostaining for cyclin E2 in the presence of cyclin E1 siRNA.** Breast cancer cells were transfected with 20nM cyclin E1 siRNA for 48h as described in [19]. Confocal images of MCF-7 cells (A.) and T-47D cells (B.) immunoprobed with cyclin E1 (red), cyclin E2 (green) and DAPI (blue). Inset at higher magnification. Experiments performed in duplicate. Scale bars = 10μm. Additional file 5:
**Representative subsets of replication-dependent and -independent histones.** Table of replication-dependent and replication independent histones used in this study. Table includes histone gene name and chromosomal location.

## Abbreviations

CDK2: Cyclin dependent kinase 2
HLB: Histone Locus Body
PLA: Proximity Ligation Assay
TCGA: The Cancer Genome Atlas
