# Supplementary material for: Cyclin E2 is the predominant E-cyclin associated with NPAT in breast cancer cells
Source: Cell Div. 2015 Feb 19;10:1. doi: 10.1186/s13008-015-0007-9 (PMC4349318; doi:10.1186/s13008-015-0007-9)
Supplement: Additional file 1: — Cyclins E1 and E2 localise to distinct nuclear foci in MCF-7 cells. A. Confocal images of MCF-7 breast cancer cells immunoprobed with cyclin E1 (red) or cyclin E2 (green), and counterstained with ToPro3 (blue, nuclei). Inset at higher magnification. Scale bars = 5 μm. Experiments are performed in triplicate. [file 13008_2015_7_MOESM1_ESM.pdf]

Additional File 1: Cyclin E1 and cyclin E2 localise to distinct nuclear foci in MCF-7 cells

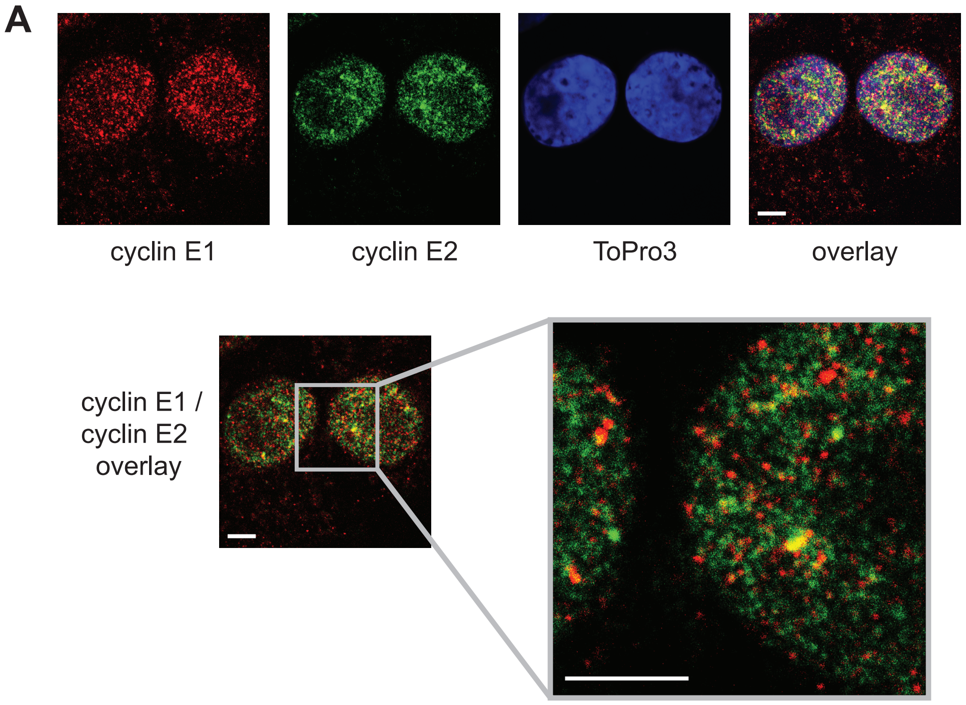

Additional File 1 – Cyclins E1 and E2 localise to distinct nuclear foci in MCF-7 cells

A. Confocal images of MCF-7 breast cancer cells immunoprobed with cyclin E1 (red) or cyclin E2 (green), and counterstained with ToPro3 (blue, nuclei). Inset at higher magnification. Scale bars = 5 μm. Experiments are performed in triplicate.
