# Supplementary material for: Cyclin E2 is the predominant E-cyclin associated with NPAT in breast cancer cells
Source: Cell Div. 2015 Feb 19;10:1. doi: 10.1186/s13008-015-0007-9 (PMC4349318; doi:10.1186/s13008-015-0007-9)
Supplement: Additional file 2: — Cyclins E1 and E2 co-sediment with centrosome components in MCF-7 cells. A. Cyclins E1 and E2 both co-purify with centrosomes. MCF-7 cells were arrested and synchronised at G0 with anti-estrogen ICI 182780 followed by estrogen stimulation for 16h. Lysates were separated by ultracentrifugation on sucrose gradients, fractionated, then pelleted and resuspended in sample buffer for western blotting with the indicated antibodies. γ-tubulin and centrin-2 are centrosome components, and Grb2 and estrogen receptor α (ER) are non-centrosomal negative controls. Data are representative of duplicate experiments. [file 13008_2015_7_MOESM2_ESM.pdf]

Additional File 2: Cyclin E1 and cyclin E2 co-sediment with centrosome components in MCF-7 cells

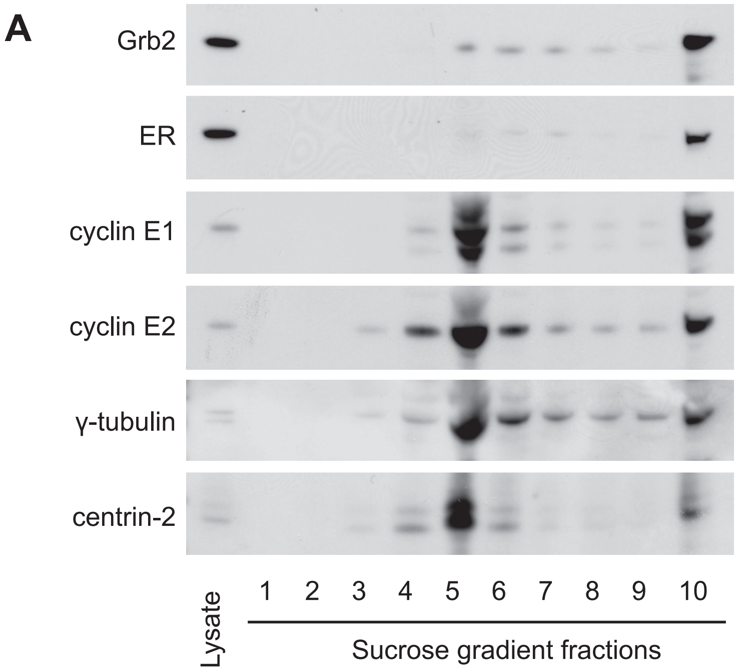

Additional File 2 – Cyclins E1 and E2 co-sediment with centrosome components in MCF-7 cells
A. Cyclins E1 and E2 both co-purify with centrosomes. MCF-7 cells were arrested and synchronised at G_0 with anti-estrogen ICI 182780 followed by estrogen stimulation for 16h. Lysates were separated by ultracentrifugation on sucrose gradients, fractionated, then pelleted and resuspended in sample buffer for western blotting with the indicated antibodies. γ-tubulin and centrin-2 are centrosome components, and Grb2 and estrogen receptor α (ER) are non-centrosomal negative controls. Data are representative of duplicate experiments.
