# Supplementary material for: Cyclin E2 is the predominant E-cyclin associated with NPAT in breast cancer cells
Source: Cell Div. 2015 Feb 19;10:1. doi: 10.1186/s13008-015-0007-9 (PMC4349318; doi:10.1186/s13008-015-0007-9)
Supplement: Additional file 3: — Cyclin E2, but not cyclin E1, co-localises with NPAT by immunofluorescence in MCF-7 cells. A. Confocal images of MCF-7 cells immunoprobed with cyclin E1 or cyclin E2 (red) and NPAT (green). Experiments performed in duplicate. Scale bars = 10μm. B. MCF-7 cells were treated with 20nM cyclin E2 siRNA for 48h as described in [19]. Co-localisation of cyclin E1 or cyclin E2 with NPAT using Pearson's correlation coefficient (PCC) which quantifies positional relationship from confocal images on a scale of -1 to +1. Statistical significance was calculated with one-way ANOVA and Tukey’s multiple comparisons, where N.S. indicates not significant and ** indicates P < 0.01. Data pooled from duplicate experiments. [file 13008_2015_7_MOESM3_ESM.pdf]

Additional File 3: Cyclin E2, but not cyclin E1, co-localises with NPAT by immunofluorescence in MCF-7 cells

A

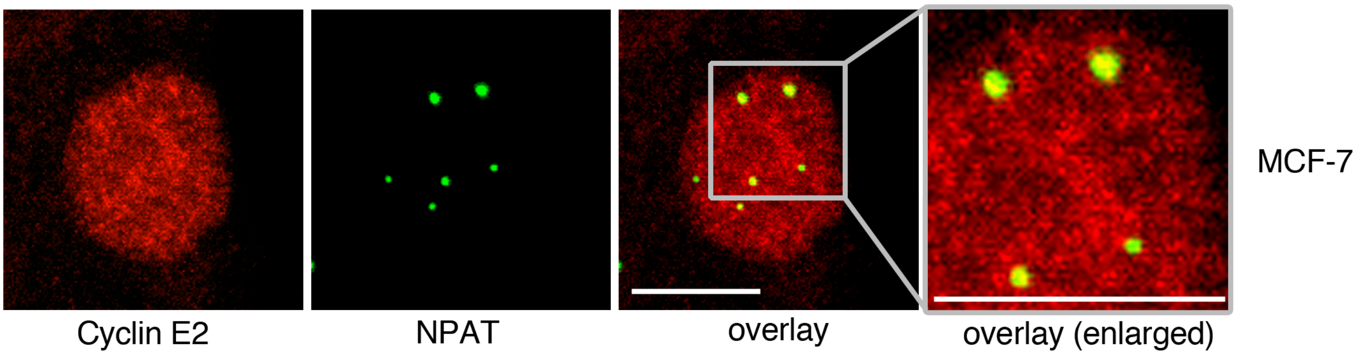

B

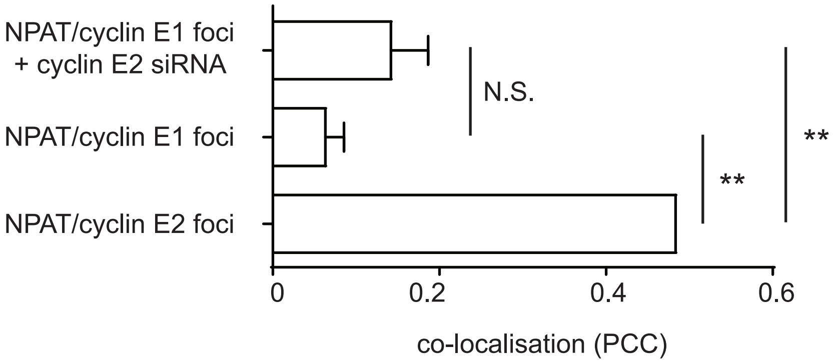

Additional File 3 – Cyclin E2, but not cyclin E1, co-localises with NPAT by immunofluorescence in MCF-7 cells

A. Confocal images of MCF-7 cells immunoprobed with cyclin E1 or cyclin E2 (red) and NPAT (green). Experiments performed in duplicate. Scale bars = 10μm. B. MCF-7 cells were treated with 20nm cyclin E2 siRNA for 48h as described in [19]. Co-localisation of cyclin E1 or cyclin E2 with NPAT using Pearson's correlation coefficient (PCC) which quantifies positional relationship from confocal images on a scale of -1 to +1. Statistical significance was calculated with one-way ANOVA and Tukey's multiple comparisons, where N.S. indicates not significant and ** indicates P<0.01. Data pooled from duplicate experiments.
