# Supplementary material for: Cyclin E2 is the predominant E-cyclin associated with NPAT in breast cancer cells
Source: Cell Div. 2015 Feb 19;10:1. doi: 10.1186/s13008-015-0007-9 (PMC4349318; doi:10.1186/s13008-015-0007-9)
Supplement: Additional file 4: — Specific immunostaining for cyclin E2 in the presence of cyclin E1 siRNA. Breast cancer cells were transfected with 20nM cyclin E1 siRNA for 48h as described in [19]. Confocal images of MCF-7 cells (A.) and T-47D cells (B.) immunoprobed with cyclin E1 (red), cyclin E2 (green) and DAPI (blue). Inset at higher magnification. Experiments performed in duplicate. Scale bars = 10μm. [file 13008_2015_7_MOESM4_ESM.pdf]

Additional File 4: Specific immunostaining for cyclin E2 in the presence of cyclin E1 siRNA

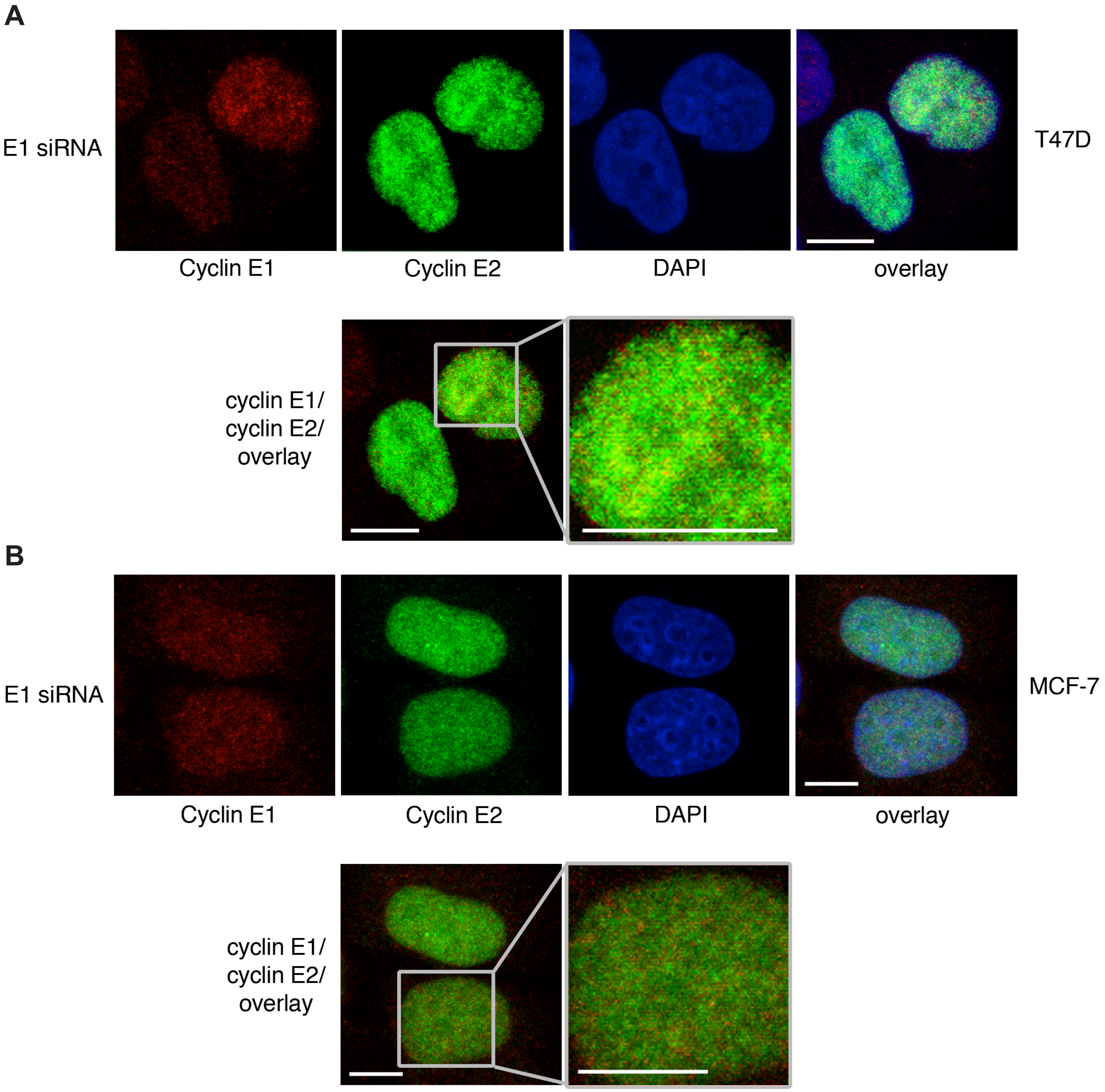

Additional File 4 – Specific immunostaining for cyclin E2 in the presence of cyclin E1 siRNA
Breast cancer cells were transfected with 20nm cyclin E1 siRNA for 48h as described in [19]. Confocal images of MCF-7 cells (A.) and T-47D cells (B.) immunoprobed with cyclin E1 (red), cyclin E2 (green) and DAPI (blue). Inset at higher magnification. Experiments performed in duplicate. Scale bars = 10µm.
