# Supplementary material for: Cyclin E2 is the predominant E-cyclin associated with NPAT in breast cancer cells
Source: Cell Div. 2015 Feb 19;10:1. doi: 10.1186/s13008-015-0007-9 (PMC4349318; doi:10.1186/s13008-015-0007-9)
Supplement: Additional file 5: — Representative subsets of replication-dependent and -independent histones. Table of replication-dependent and replication independent histones used in this study. Table includes histone gene name and chromosomal location. [file 13008_2015_7_MOESM5_ESM.pdf]

Additional File 5: Representative subsets of replication-dependent and -independent histones

Histone Class	Name	Chromosome
Replication-dependent histones	HIST1H2AG	6p22.1
	HIST1H2AH	6p22.1
	HIST1H2BB	6p22.1
	HIST1H2BF	6p22.1
	HIST1H4J	6p22.1
	HIST1H4L	6p22.1
Replication-independent histones	H1F0	22q13.1
	H1FX	3q21.3
	H2AFJ	12p12.3
	H2AFX	11q23.3
	H3F3A	1q42.12
	H3F3B	17q25.1
